# Risk prediction models for erosive wear in preschool-aged children: a prospective study

**DOI:** 10.1186/s12903-022-02334-8

**Published:** 2022-07-28

**Authors:** Gabriella Gatt, Nikolai Attard

**Affiliations:** 1 Department of Child Dental Health and Orthodontics, Faculty of Dental Surgery, University of Malta, Msida, Malta; 2 Department of Oral Rehabilitation and Community Care, Faculty of Dental Surgery, University of Malta, Msida, Malta

**Keywords:** Erosive tooth wear, Preschool aged children, Basic Erosive Wear Examination (BEWE) Index, Risk prediction model, Primary dentition

## Abstract

**Background:**

Despite increasing prevalence, age-specific risk predictive models for erosive tooth wear in preschool-age children have not been developed. Identification of at-risk groups and the timely introduction of behavioural change or treatment will stop the progression of erosive wear in the permanent dentition. This study aimed to identify age-specific risk factors for erosive wear. Distinct risk prediction models for 3-year-old and 5-year-old children were developed.

**Methods:**

A prospective cohort study included school-based clinical examinations and parent administered questionnaires for consented 3 and 5-year-old healthy children. Calibrated examiners measured the following health parameters under standardised conditions: erosion, using the Basic Erosive Wear Examination Index (*BEWE*), caries using the International Caries Detection and Assessment System (*ICDAS*), plaque and calculus according to the British Association for the Study of Community Dentistry (*BASCD*) scores, dental traumatic injuries and soft tissue lesions, salivary testing and BMI. Other health conditions were collected via a parent-administered questionnaire that explored oral- and general-health. Non parametric tests were utilised to explore the temporal relation of erosion with, demographic factors, oral hygiene habits, general health and dietary habits. Variables showing significance with a difference in BEWE cumulative score over time were utilised to develop two risk prediction models. The models were evaluated by Receiver Operating Characteristics analysis.

**Results:**

Risk factors for the 3-year-old cohort (N = 336) included erosive wear (χ^2^(1, 92) = 12.829, p < 0.001), district (χ^2^(5, 92) = 17.032, p = 0.004) and family size (χ^2^(1, 92) = 4.547, p = 0.033). Risk factors for the 5-year-old cohort (N = 441) also included erosive wear (χ^2^(1, 144) = 4.768, p = 0.029), gender (χ^2^(1, 144) = 19.399, p < 0.001), consumption of iced tea (χ^2^(1, 144) = 8.872, p = 0.003) and dry mouth (χ^2^(1, 144) = 9.598, p = 0.002).

**Conclusions:**

Predictive risk factors for 3-year-old children are based on demographic factors and are distinct from those for 5-year-old children based on biological and behavioural factors. Erosive wear is a risk factor for further wear in both age cohorts.

## Introduction

The spectrum of tooth surface loss (TSL) is made up of the different types of tooth wear [1] excluding those caused by caries and trauma. While historically abrasion was the main contributor to TSL, aetiology shifted to a more even contribution from abrasion, attrition and erosion [2] and more recently erosive wear is being recorded as the most dominant of the three wear processes and as that with increasing prevalence in all ages [3]. Erosion is the acid demineralisation of hard tooth tissue that enhances the mechanical wear caused by attrition and abrasion. The three act synergistically.

International clinical studies carrying out partial mouth recordings in preschool aged children have reported prevalence figures for erosion ranging from 5.7% in China [4, 5], 29% in India [6, 7], 31% in Saudi Arabia [8], 47% in Ireland [9], and 51% in Brazil [10]. Clinical studies carrying out full mouth recordings have reported prevalence figures ranging from 0.6% in Brazil [11], 30% in Germany [12], 57% in the United Kingdom [13], 52% and 79% in Greece [14–16], 77% in Australia [17] and 80% in 5 year old children in Norway [18]. Two previous longitudinal studies on the prevalence of erosive tooth wear in a cohort of the preschool children have been carried out [10, 19]. While one study reported no increase in prevalence or severity over a 4 year period in Brazilian children aged 3–4 years old [10], the other study reported an increase from 0 to 28% over a period of 2 years in 2–4 year old Australian children [19].

Research suggests that erosive lesions in the primary dentition are a predictive factor for erosive tooth wear in the permanent dentition [20]. Children presenting with erosive lesions at age five, are 5 times more likely to present with erosive lesions at age twelve [21]. When still in its early stages of development, erosive tooth wear is difficult to diagnose [22] and its effect on the oral health related quality of life of the patient is not clear [23]. Yet when allowed to progress, erosive lesions in the primary dentition are a challenge to treat as successful adhesive restorations are limited by patient compliance, inadequate enamel and insufficient coronal tissue and are most times left untreated [24]. Tooth wear in the primary dentition should not be overlooked as erosive wear may have lifetime consequences upon the child’s dentition. The provision of complete dental care to the child patient population therefore warrants the diagnosis of erosive wear, the identification of the aetiological factors and the implementation of preventive measures.

Studies testing individual predictors in isolation is no guarantee of their true predictive role [25]. Lussi and Jaeggi [26] state that bivariate analyses of the correlation between the chemical properties of individual products and their erosive potential alone can be misleading. What must be determined is how identified risk factors relate to other known risk factors in the overall aetiology of the condition. Multivariate modelling allows for this analysis of the simultaneous effects of multiple variables and the generation of prediction models. These guide healthcare workers by supplementing clinical decisions by providing objective probabilities that particular outcomes will occur in the presence of delineated sets of predictors. Personalised preventive or interventional treatment may then be withheld, initiated or lifestyle changes recommended depending upon personal levels of risk [25, 27]. Such research has been carried out and risk models for other dental conditions of multifactorial aetiology such as dental caries [28–30], periodontal disease [31, 32] and craniofacial conditions [27] have been developed.

Such risk models are useful in the COVID-19 pandemic context when non-emergency dental visits have declined, periodicity of dental check-ups has been disrupted [33] and the increased time spent at home has been associated with non-ideal dietary habits [34]. Early identification of those at risk and the opportunity to introduce behavioural advice or intervention in a timely fashion has become more important. No risk model for erosive dental wear in the preschool aged child has yet been developed.

The aim of this study was to develop risk prediction models for erosive dental wear in preschool aged children. The purpose of the models is to guide general dental practitioners in identifying those paediatric patients at risk of progressive erosive wear into their permanent dentition. The null hypothesis tested was that there is no difference in a prediction model developed for a 3-year-old population and a 5-year-old population of children.

## Materials and methods

### Study design

Three- and five-year-old children attending pre-school were invited to participate in a prospective cohort study including a clinical examination and a parent questionnaire. A multi-stage cluster sampling technique was utilised in order to ensure proportionate representation of all localities of residence, school types and socio-economic bands.

### Setting

This study was carried out in a central Mediterranean island consisting of an archipelago of five islands with a population of just over 400,000. The climate is characterised by hot summers and mild winters with monthly average temperatures ranging from 15 to 31 °C in the summer months.

A detailed research protocol was prepared abiding to all the requirements as stated in the World Medical Association Declaration of Helsinki – Ethical Principles for Medical Research Involving Human Subjects, WMA General Assembly, 2008. The protocol was submitted for consideration, guidance and approval to the Faculty of Dental Surgery Research Ethics Committee and subsequently to the University of Malta Research Ethics Committee [UREC MD 31/2013]. The study was also registered with the Local Data Protection Officer.

Approval was also sought from the relevant authorities in the three school streams. (State schools, Church schools and Independent schools). Additional signed parental consent was also sought after having distributed information sheets to all parents/legal guardians at least three weeks prior to the school visit.

### Calibration of examiners

Training and calibration of examiners and scribes in the use of the BEWE Index [35] and in periodontal diagnosis and charting was carried out by internationally renowned researchers in the fields. The examiners included four dental surgeons; the scribes were two dental hygienists. Training and calibration programmes organised by the Faculty of Dental Surgery, University of Malta, included seminars, discussions, simulation lab sessions and clinical sessions over several days. Training in the use of the International Caries Detection and Assessment System (ICDAS) was carried out individually via the eLearning programme portal provided by the ICDAS Foundation [36]. Further calibration sessions were carried out involving duplication of examination of clinical cases in order to assess intra- and inter-examiner reliability.

### Clinical examination

Examinations were held on school premises during school hours. A portable dental unit (D-13600 Denta-Trolley, BPR Swiss-Switzerland) provided compressed air. A Daray X200LED mobile examination light provided a standardised source of light delivering 8000 lx at 1 m and 32,000 lx at 0.5 m (Daray Lighting Ltd., Leighton Buzzard, Luton, UK). Individually wrapped sterile packs containing a front surface reflecting mirror and a ball-ended World Health Organization CPITN-E (Community Periodontal Index of Treatment Needs) probe were available for each participant. Data were recorded by trained scribes onto number coded data input sheets. The children were examined in a supine position by examiners wearing personal protective equipment. Repeat examinations of two randomly selected children were carried out at each school visit to check intra-examiner reproducibility. Participants were screened for erosive tooth wear and dental caries. They were also charted for the presence of plaque, calculus, dental traumatic injuries and soft tissue lesions. Each child needing treatment was given a referral note.

### Participants

The selection criteria included:

- Inclusion criteria: those children resident on the Islands all their lives and who turned 3 or 5 years old in that calendar year.
- Exclusion criteria: Children exhibiting enamel defects associated with loss of tooth tissue. Children who did not return a signed consent form were also excluded.

The 3-year-old cohort, identified as the (3–5) group, was re-examined 2 years later while the 5-year-old cohort, identified as the (5–8) group was re-examined 3 years after the initial screening visit. The difference in the screening time was due to the restricted number of examiners and scribes involved in this project.

### Variables

The clinical examination recorded erosive tooth wear, dental caries, oral hygiene level, salivary parameters and Body Mass Index (BMI).

*Erosive tooth wear*—The BEWE Index was used to score an index value per participant [35]. The BEWE Index examines all surfaces of all teeth (excluding third molars) and records the highest score (0–3) for each sextant which scores then contribute to the individual’s cumulative score (0–18). For the purpose of this study, participants were assigned to an erosion experience category according score: BEWE 1—scores 0–2, no risk; BEWE 2—scores 3–8, low risk; BEWE 3—scores 9–18, medium—high risk.

Outcome measurement was always blinded to or independent of any knowledge of the predictors under consideration.

*Dental caries*—ICDAS [36] was utilised as a system for detecting and classifying carious lesions present.

*The BASCD Plaque Score* employed was that according to the BASCD (British Association for the study of Community Dentistry) [37] criteria. Scores for each tooth were as follows: 0—no plaque present, 1—little plaque visible on probing, 2—substantial amount of plaque visible to the naked eye, 9—no assessment possible. Calculus was scored as either 0—no calculus present or 1—calculus present.

*Salivary parameter testing* Salivary testing was carried out using the Saliva-Check BUFFER™ kit (GC Corp., USA). This test assessed the participants’ salivary flow, pH and buffering capacity. Provided manufacturer instructions were followed. Samples were taken during the above-described clinical examination. Unstimulated salivary flow rate was measured by everting the lower lip, blotting it dry with tissue and observing the mucosa under good light. The timing for the formation of droplets of saliva to appear at the orifices of the minor salivary glands was noted. If droplets took less than 60 s to appear, salivary flow was recorded as ‘normal’. If droplets took more than 60 s to form salivary flow was recorded as ‘slow’. Buffering capacity was assessed by removing a Buffer test strip from the foil packaging and placing on an absorbent tissue with its test side up. Using the pipette provided, sufficient saliva was drawn from the patient’s mouth and a drop of saliva was dispensed on each of the three test pads. The strip was immediately turned 90º onto its side to allow excess saliva to flow onto the absorbent tissue. This was done to prevent excess saliva from swelling the test pad and possibly affecting the accuracy of the result. At the end of the clinical examination the test pad was evaluated and the buffering capacity result was calculated by referring to the conversion table provided, and adding up and the points according to the colour change of each pad. A combined total score of 0–5 was recorded as a Very Low buffering capacity, a score of 6–9 was recorded as a Low buffering capacity and a score of 10–12 was recorded as a Normal/High buffering capacity. Salivary pH was measured by dispensing a drop of saliva on a pH test strip provided and allowing it to rest for 10 s. The colour change of the strip was compared with the testing chart made available and a reading was taken as either highly acidic (pH 5.0–5.8), moderately acidic (pH 6.0–6.6) or healthy saliva (pH 6.8–7.8).

*Height and weight measurements—BMI* Anthropometric measurements were carried out and recorded using a portable stadiometer (SECA 214 portable Stadiometer) and portable digital scales (SECA 875 flat scales). Children were asked to remove their school shoes and were measured wearing their uniforms. Participants were instructed to hold the Frankfort plane parallel to the ground during measurements. BMI was then calculated as weight divided by the square of the height (Kg/m^2^). The calculated BMI values were divided into four categories (Thinness, Normal, Overweight and Obesity) according to the International Obesity Task Force cut-off values [38]. Cut-off points at the mid-year value (3.5 years and 5.5 years) were utilised as recommended when carrying out epidemiological studies including age groups of one year width [38].

*Parent questionnaires* A piloted and sequentially refined questionnaire in both English and Maltese was distributed to parents/legal guardians of all participants. Instructions were clear and an example was provided. A combination of both closed and open-ended questions were included. The response category ‘don’t know’ was included where appropriate. Responses for closed questions were converted to numerical format and responses to open-ended questions were summarised into categories. This allowed for entry into IBM SPSS and statistical analysis.

The questionnaire was designed to enquire about predictors for erosive wear as identified by literature review. These included questions about sociodemographic factors, oral health habits, dietary habits and general health factors. Sociodemographic variables included gender, age, locality and duration of residence, parental educational level (divided into 4 levels: Primary School level, Secondary School level, Post-Secondary School level and Tertiary level), and parental job type (4 subdivisions: Professional, Clerical/Business, Manual labourer and Unemployed). Oral hygiene related questions enquired about the frequency (never, less than once a day, once a day, two or more times a day) and timing (before breakfast, after breakfast, after lunch, before bed and other times) and technique of brushing (horizontal, vertical, circular or a combination of all), the type of brush (manual or electric) used, type of toothpaste (adult with fluoride, adult without fluoride, children’s with fluoride, children’s without fluoride, whitening toothpaste) and amount of toothpaste used (smear, smaller than pea-sized, pea-sized full length of toothbrush head or over flowing) and whether the child rinses after brushing. Dietary habits related questions included questions related to the drink consumed most on a daily basis and after sports activities and at night, the temperature of the drink, swallowing habits (swishing, retaining in the mouth, none), timing of drinking (sipping over a period of time, in one episode) and the use of a straw. Parents were then presented with a chart listing twenty-eight food and drink items and were asked to select the frequency of consumption of their child for each item on a scale of ‘more than four times a day’, 2–4 times a day’, ‘once a day’, ‘1–2 times a week’, ‘less than once a week’ and ‘never’. General health related questions enquired about use of medications, asthma and its treatment, hospitalisation, gastro-oesophageal reflux disease, history and duration of vomiting and symptoms of dry mouth. The distributed questionnaires were number coded per participant to match the data input forms. An identical copy of the questionnaire was redistributed to the same cohorts at the follow-up visit. The questionnaires were distributed and re-collected by school administration staff.

### Study size

A review of the pertinent literature analysed prevalence data figures for dental erosion in this age group. This was done in order to estimate the expected prevalence and thereby guide sample size calculations. The statistical package Epi-Info™ recommended a sample size of 351 3-year-old children and 349 5-year-old children. This was in accordance with total national populations of 4026 3-year-old and 3788 5-year-old children respectively, a degree of accuracy of p = 0.05 and a confidence level of 95%. Deliberate over-sampling was carried out in anticipation of study cohort attrition due to factors including non-compliance from pre-cooperative participants, absentees, refusals of consent and failure of returned questionnaires. Sample sizes of 400 3-year-old and 420 5-year-old children were identified.

### Statistical methods

Tests for normality were run for all the variables. The data collected were both of a continuous and categorical nature. Descriptive statistics were carried out in order to describe the characteristics of the sample including Average Cumulative scores, percentage scores of BEWE experience categories and incidence figures. The Chi-square test for independence was used to explore the relationship between the frequencies for categorical demographic variables scored.

The Wilcoxon signed rank test was conducted to evaluate the temporal effect on the frequencies of consumption of dietary constituents (2 years in the (3–5) cohort and 3 years in the (5–8) cohort). The relationship between the change in BEWE scores and the change in food frequencies was investigated using the Spearman Rank Order correlation test. A Mann–Whitney U test was conducted to compare the change in BEWE scores over time for the 26 bivariate categorical variables including age, gender, asthma diagnosis, reflux symptoms and grinding habits. Kruskal–Wallis tests allowed comparison of scores of changes in BEWE scores over time across the remaining 55 categorical variables representing demographic factors, oral hygiene habits and dietary habits. The relationship between change in BEWE scores and all the continuous variables was investigated using Spearman Rho correlation coefficient Test. These variables included results for BMI, number of siblings, deft scores, buffering capacity scores and salivary pH.

The dependent variable was the increment in erosive tooth wear in the preschool aged child. Risk indicators/factors and predictor variables were employed, as allowed by risk prediction models. This was done in an effort to maximise sensitivity and specificity. Risk indicators/factors included demographic data (age and gender), behavioural data (oral hygiene practices and dietary habits), and biologic factors (salivary flow/buffering capacity). Predictor variables included baseline BEWE scores for erosive tooth wear and baseline deft scores for caries diagnosis.

Two prognostic prediction models were developed from the data collected from this prospectively collected cohort. The models were developed using the Generalised Linear Model function—one for each age cohort. This allowed the simultaneous analysis of multiple variables including a mixture of categorical and continuous variables. Those variables that showed a statistically significant relationship with a difference in BEWE cumulative score over time together with those up to a significance level of p = 0.1 were utilised to develop two risk prediction models. A forward procedure was performed adding and retaining predictors with p < 0.05 in the model. The sensitivity and specificity of the model was calculated and the model was evaluated by ROC analysis. A BEWE cumulative score of 9 was taken as a cut-off value.

Statistical tests were carried out using SPSS 20.0 software (IBM Company, Chicago, IL, USA). Statistical significance for all tests was set at p < 0.05.

## Results

### Participants

**Fig. 1:**
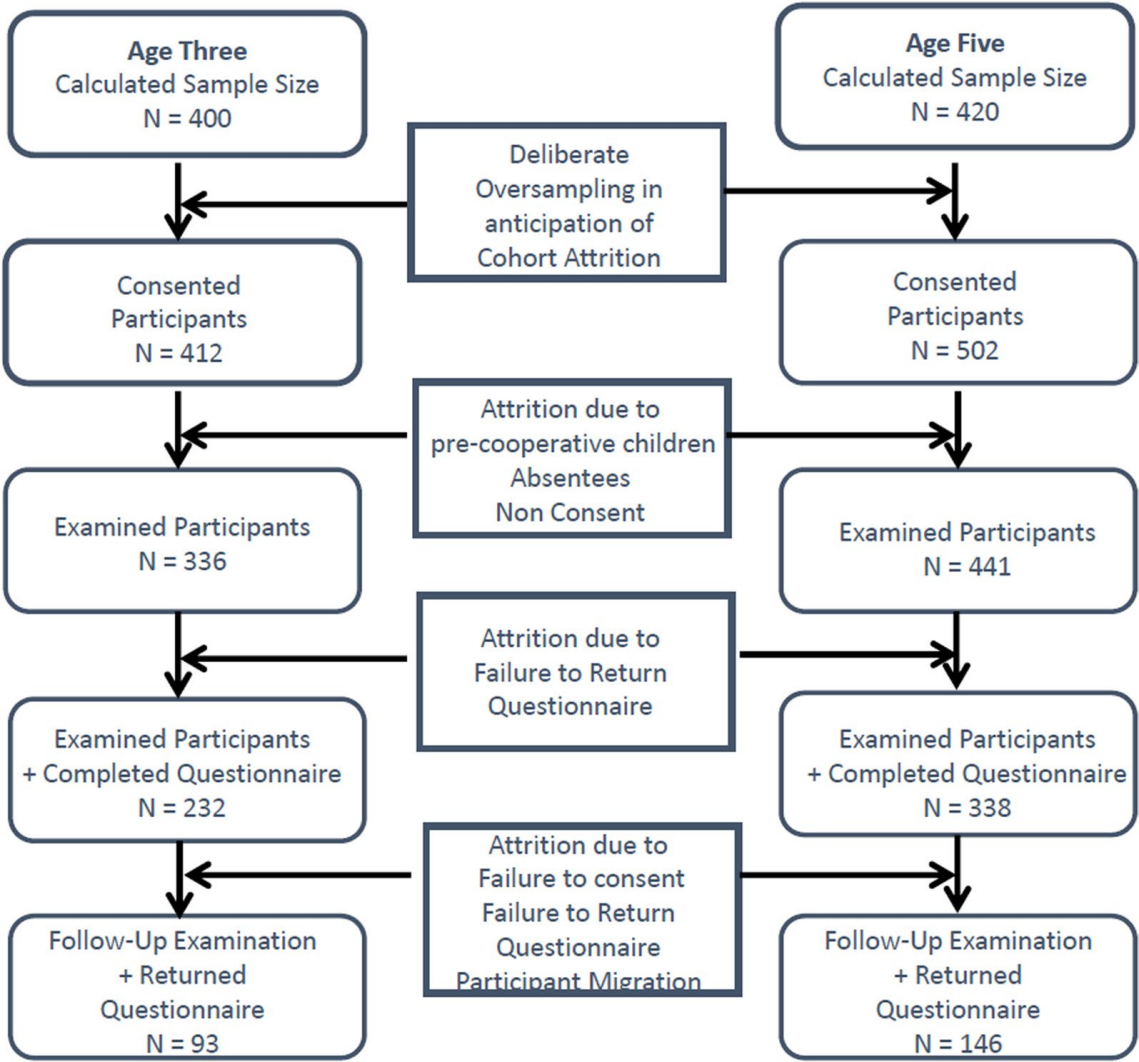
Flow diagram depicting sample numbers

The study participants included in the study included a cohort of 3-year-old children and 5-year-old children as described in Fig. 1. With 19 participating schools, 239 children were screened again at follow-up including 139 males and 100 females. In the (3–5)-year-old cohort the response rate to participate in the follow-up screening was 59% (N = 138) of the original examined cohort who returned the questionnaire (N = 232). The questionnaire response rate in this group was then 67% (N = 93). In the (5–8)-year-old cohort the response rate to participate in the follow-up screening was 57% of the original examined cohort who returned the questionnaire (N = 193). The questionnaire response rate in this group was then 76% (N = 146).

The (3–5)-year-old cohort were screened at follow-up in one calendar month, 2 years after the initial screening. The (5–8)-year-old cohort were screened at follow-up in one calendar month, 3 years after the initial screening.

The data for the (3–5)-year-old cohort were analysed independently of the data for the (5–8) year-old cohort.

Kappa values for intra-examiner reproducibility were 0.86 for the BEWE Index, 0.82 for the ICDAS Index and 0.90 for the BASCD Plaque Score Index. Inter-examiner reproducibility tests rendered values of 0.79 for the BEWE Index, 0.85 for the ICDAS index and 0.80 for the BASCD Plaque Index.

Interpretation of the skewness and kurtosis and the Kolmogorov-Smirov statistics indicated that the data were not normally distributed.

There was no difference between the demographic profile and socioeconomic profile of the baseline and follow-up cohorts for gender, districts, school type, parental jobs and BEWE scores (chi-square test), the result was not significant at p < 0.050.

### Erosive tooth wear prevalence, incidence, distribution and severity

Table 1 presents the results comparing the data at baseline to that at the follow-up phase. Figures denote the increase in prevalence, spread and severity with time.

**Table 1:** Erosion experience, severity and distribution

	Age three		Age five	
	Baseline T0	Follow-up T1	Baseline T0	Follow-up T2
	N = 336	N = 93	N = 441	N = 146
Mean BEWE cumulative score (sum of scores of all the sextants)	3 SD 3.56 95% CI 2.62–3.37	8.5 SD 5.2 95% CI 7.46–9.54	4 SD 3.79 95% CI 3.65–4.35	8.8 SD 5.56 95% CI 7.9–9.7
Participants with a cumulative score > 9	8% (N = 29)	42% (N = 39)	13% (N = 56)	46% (N = 67)
Signs of erosive wear on at least one tooth surface (BEWE cumulative score > 0)	71% (N = 236)	99% (N = 92)	81% (N = 356)	92% (N = 135)
Mean number of sextants affected	2.11 SD 2.144 95% CI 1.85–2.37	4.2 SD 1.72 95% CI 3.85–4.55	2.43 SD 2.06 95% CI 2.21–2.649	4.26 SD 1.79 95% CI 3.97–4.55
BEWE score 3 in upper labial segment	16%	60%	19%	29%

In the (3–5)-year-old-cohort 81% (N = 75) of children developed new erosion.

The incidence proportion was 75/93 = 0.8

The mean change BEWE Cumulative score was 5.6 (SD 5.2).

In the (5–8)-year-old-cohort 77% (N = 112) of children developed new erosion.

The incidence proportion was 112/146 = 0.8

The mean change BEWE Cumulative score was 5.7 (SD 4.8).

Table 2 presents the difference in distribution of scores of BEWE categories for both age cohorts at baseline and then again at follow-up.

**Table 2:** Percentage change in BEWE scores in relation to baseline BEWE score

Cumulative score	BEWE erosion experience categories for this study	Age three N = 93			Age five N = 146		
		Baseline	Follow-up	Δ BEWE score	Baseline	Follow-up	Δ BEWE score
≤ 2	**BEWE 1**	51% (N = 47)	10% (N = 9)	− **80%**	40% (N = 59)	16% (N = 24)	− **60%**
3–8	**BEWE 2**	41% ( N = 38)	48% (N = 45)	**+ 17%**	43% (N = 62)	38% (N = 55)	− **11.6%**
9–13	**BEWE 3**	6% (N = 6)	23% (N = 21)	**+ 283%**	14% (N = 20)	21% (N = 30)	**+ 50%**
≥ 14		2% (N = 2)	19% (N = 18)	**+ 850%**	3% (N = 4)	25% (N = 37)	**+ 733%**

Bold: a reduction in the number of scores of BEWE 1 ( least severe) were observed and an increase in the number of the scores signifying more severe erosive wear ( BEWE 2 and BEWE 3) were observed

#### Risk predictors significantly correlated with change in BEWE scores—(3–5) year old cohort

The variables found to be correlated with a Change in BEWE score were the baseline BEWE cumulative score (rho = -0.384, n = 93, p < 0.005), follow-up BEWE cumulative score (rho = 0.680, n = 93, p < 0.005), number of siblings (rho = -0.031, n = 93, p = 0.002), bruxism habit ((Md = 7, n = 15) (Md = 3, n = 78), U = 366.5, z = -2.297, p = 0.002), frequency of consumption of fresh fruit (rho = 0.225, n = 92, p = 0.031) and consumption of sour foods ((Md = 8, n = 5) (Md = 3, n = 88), U = 97.0, z = -2.108, p = 0.035).

#### Risk predictors significantly correlated with Change in BEWE scores—(5–8) year old cohort

The variables found to be correlated with a Change in BEWE score were follow-up BEWE cumulative score (rho = 0.731, n = 146, p < 0.005), gender ((Md = 3, n = 59) (Md = 6, n = 87), U = 1734.5, z = -3.325, p = 0.001, p = 0.001), BMI score (rho = 0.205, n = 146, p = 0.004) and frequency of consumption of iced tea (rho = 0.23, n = 143, p = 0.050).

These variables together with those that reached a significance level of 0.1 were utilised to develop two risk prediction models. The models were then evaluated using ROC curves.

### Risk prediction model for (3–5)-year-old cohort

**Fig. 2:**
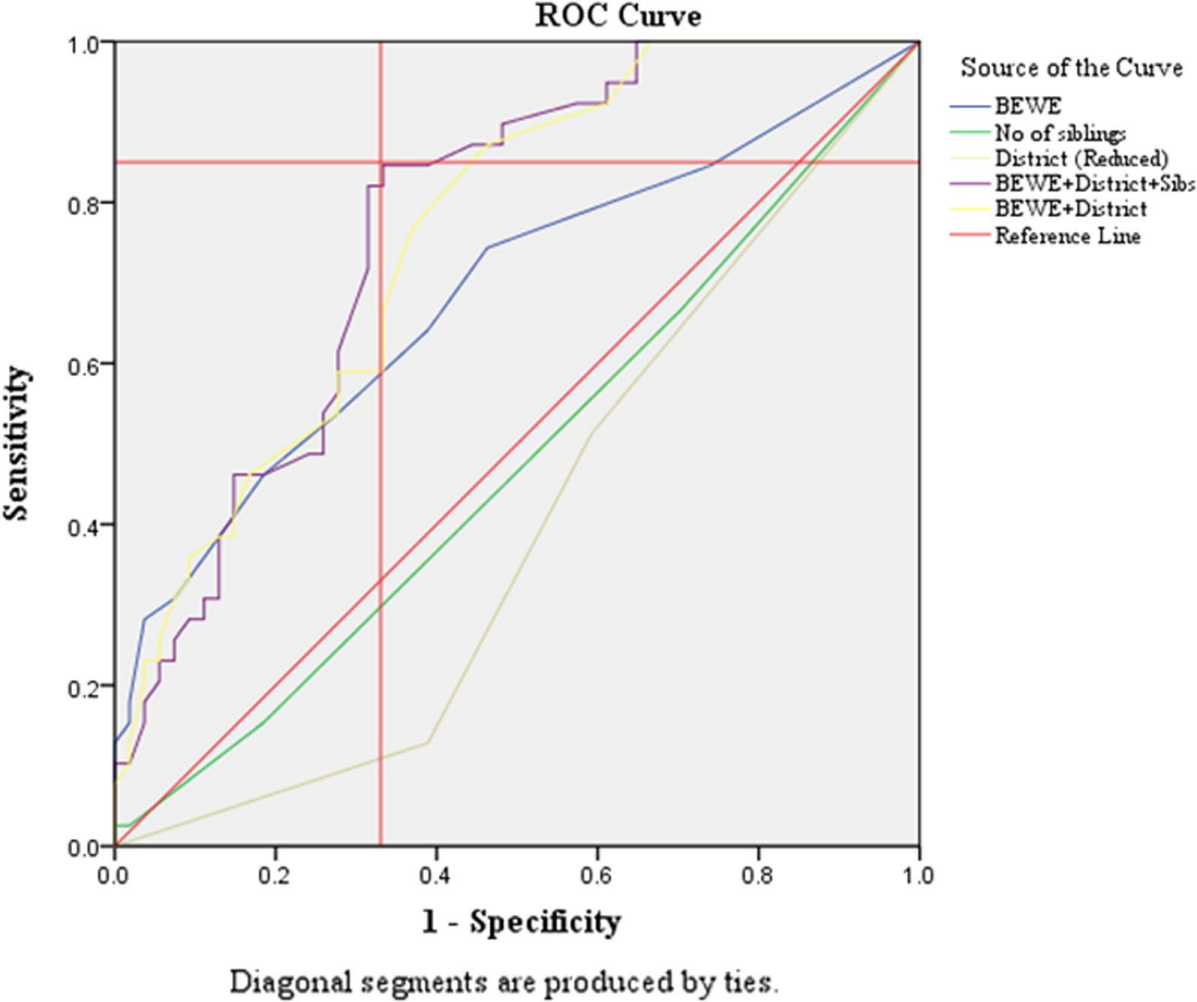
ROC curve for risk predicton model for the 3-year-old cohort

ROC curves were generated including a variety of combinations of the selected variables. The variables utilised to achieve the curve with the greatest area under the curve (AUC) (0.8) (SE 0.048, 95% CI 0.676–0.864, p < 0.001) included the three variables Baseline BEWE (Wald χ^2^(1, n = 93) = 12.829, p < 0.001), district (Wald χ^2^ (5, n = 93) = 17.032, p = 0.004) and number of siblings (Wald χ^2^(1, n = 93) = 4.547, p = 0.033). Figure 2 illustrates how the point at which it reached its greatest distance from the reference line is that at a sensitivity of 85% and specificity of 70%. This is associated with a likelihood ratio for a positive result of 2.8 when using this predictive model.

### Risk prediction model for (5–8)-year-old cohort

**Fig. 3:**
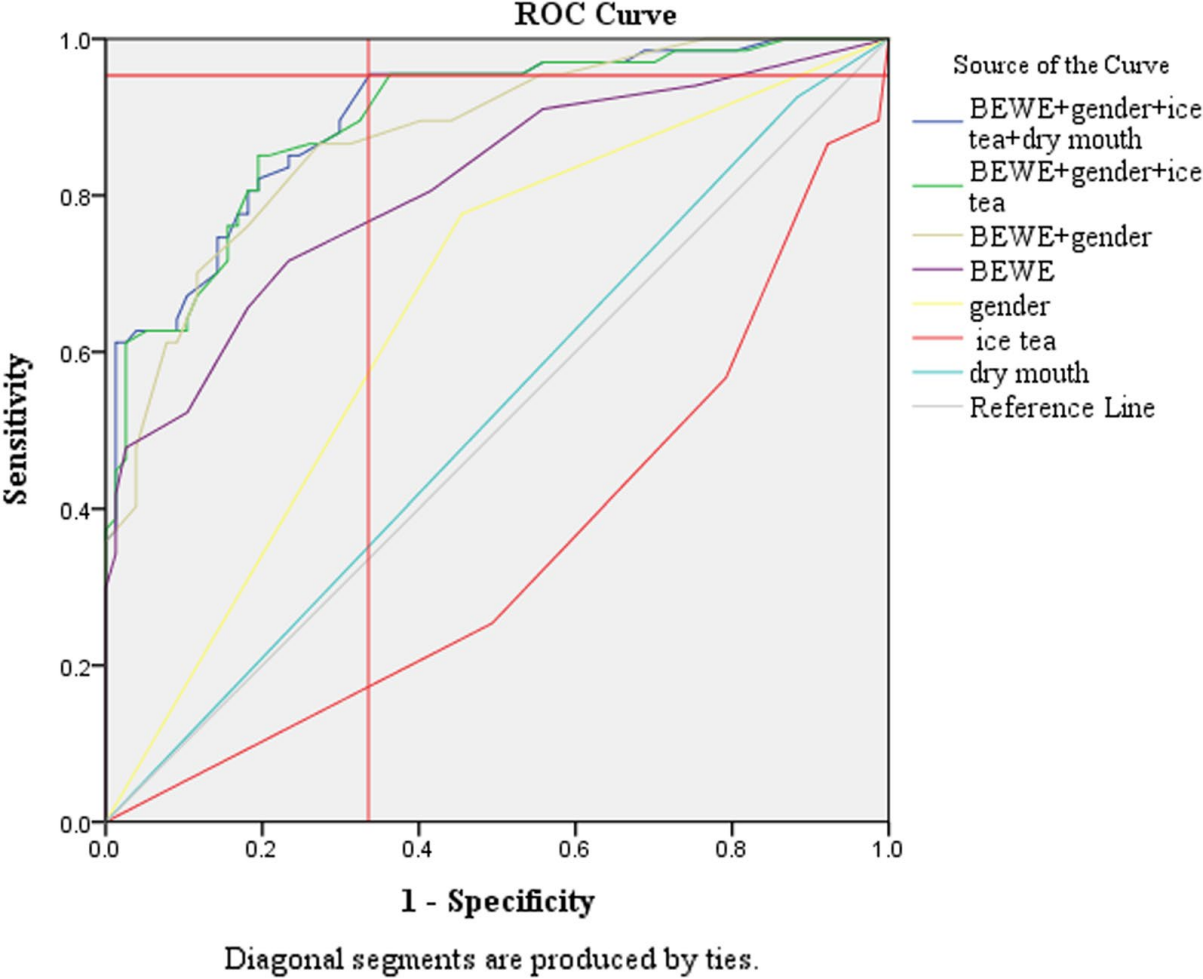
ROC curve for risk predicton model for the 5-year-old cohort

ROC curves were again generated including combinations of the significant variables for this age cohort. The curve with the greatest AUC (0.9) (SE 0025, 95% CI 0.850–0.949, p < 0.001) was that of the model that included the variables Baseline BEWE (Wald χ^2^(1, n = 146) = 9.958, p = 0.002), gender (Wald χ^2^(1, n = 146) = 19.399, p < 0.001), consumption of ice tea (Wald χ^2^(1, n = 146) = 8.872, p = 0.003) and complaint of dry mouth (Wald χ^2^(1, n = 146) = 4.768, p = 0.029). Figure 3 illustrates how the point at which it reached its greatest distance from the reference line is that at a sensitivity of 95% and specificity of 67%. This is associated with a likelihood ratio for a positive result of 2.9 when using this predictive model.

## Discussion

This longitudinal study examined two cohorts of preschool aged children aged three and five at two separate intervals 2 and 3 years apart respectively. At each intervention, a clinical examination and a parent questionnaire collected data relating to erosive wear, dental caries, periodontal health, demographic and socioeconomic factors together with dietary habits and oral hygiene practices. An increase in the incidence, prevalence, severity and extent of erosive tooth wear in both the three and five aged cohorts over time was reported. The data collected were utilised to create risk prediction models for each age group and to reject the hypothesis that there is no difference in the risk models developed for the two age groups.

### Progression of erosive wear

The high prevalence of erosive tooth wear and the observed increase in severity and extent observed over time in this study is in accordance with the findings of previous literature that also express concern that the condition is increasing particularly in younger populations [10, 39, 40]. Both cohorts demonstrated a linear association of wear with age. Additionally, both cohorts also demonstrated, an increase in severity of wear that was greater with higher initial BEWE cumulative scores. Having erosive wear therefore predisposes to a greater rate of further erosive wear. Additionally, this study highlights that prevalence and severity rates per age group are increasing progressively. This is seen in Table 1, where 81% of the 5-year-old children at time T0 had as BEWE Cumulative Score > 1, with a mean of 4, 13% of which scored > 9 and exhibited a mean of 2.4 sextants affected. In the 3-year-old cohort examined at time T1, 2 years later at age 5, 99% had as BEWE Cumulative Score > 1, with a mean of 8.5, 42% of which scored > 9 and a mean of 4.2 sextants affected.

These findings indicate that dietary habits and patterns of consumption are in flux, that once erosive wear sets in, unless directed efforts are made, the erosive process will proceed, as habits picked up at young ages tend to stay with the person. It is therefore important that the key predictive factors for erosive wear be identified in order to guide clinical decision making into introducing primary and secondary preventive measures at the high-risk preschool age children.

#### Risk predictive models

Longitudinal studies are the recommended source of data for the development of a prediction model [25, 41] as they satisfy the criteria necessary for identifying risk factors [27]. Both models, prognostive in nature, while not a substitute for clinical experience, were intended to provide objective data, inform decision-making and help overcome bias [41]. The models were evaluated by ROC analysis for which BEWE cumulative score of 9 was taken as a cut-off value. BEWE Index cumulative score bands are linked to treatment recommendations with patients scored 9 and above considered to be at moderate to severe risk of erosive tooth wear requiring more than just observation and routine maintenance. This defined the participants classified as exhibiting the outcome of interest and ensured that the models were to be clinically relevant.

### The (3–5) year old cohort

The two-year study period for this cohort allowed for observation of the primary dentition from time of full eruption to before the early mixed dentition stage. The significant attrition of numbers in this group was due to children moving on from nursery schools into primary school. This cohort experienced an incidence proportion of 0.8 and an increase in prevalence, severity and extent of erosive tooth over 2 years. Participants with a BEWE Cumulative score of 9 or more increased from 8.0 to 42% by the age of five. Similar to previous research [10, 42–45] the upper labial segment was the site mostly affected. Involvement of this sextant increased from 16% at 3 years of age to 60% 2 years later. This could be either due to its earlier time of eruption compared to the rest of the dentition or due to its exposed location at the front of the mouth.

#### Risk predictive model—age 3

The final risk model for this age cohort included three erosive wear related predictors: baseline BEWE score (p < 0.001), district (p = 0.004) and number of siblings (p = 0.033). This is a model with an AUC of 0.8 meaning that it able to predict cases in 80% of times.

This study corroborates previous research that the presence of erosive wear carries a risk for further erosive wear [20, 21]. Baseline BEWE alone was also able to deliver a risk predictive model with and AUC of 0.7. This indicates that once erosion sets in in a mouth, unless directed efforts are made to change contributory factors the likelihood is that the erosive process shall proceed. The number of siblings in the family was inversely related to change in BEWE cumulative score (SE 0.6032, 95% CI − 2.468 to − 0.104). This could be interpreted to show that the smaller the family size the greater availability of income for spending on erosive drinks rather than water and milk. The districts with the highest percentage of persons living at-the-risk-of-poverty, as indicated by the National Statistics Office, Malta, 2021 [46] had a mean value for change in BEWE cumulative score of 7.3. In contrast regions, reported to have the lowest at-risk-of poverty indicators, displayed the lowest mean change in BEWE cumulative score value of 2.5. The study of the influence of socioeconomic status on erosive wear has given conflicting results in the literature. A systematic review by Kreulen [47] reports a positive correlation with economic status in six studies and an inverse relationship in seven studies with no effect in another two. Such a variable would however be expected to vary between cultures and generations on a global scale. These findings clearly indicate that the risk of erosive tooth wear in the 3-year-old child is highly influenced by sociodemographic factors and is therefore a health outcome affected by social determinants. Exploring the possible influences on the determinants of erosive wear by family, community and socio-cultural systems is an area for further research.

#### The (5–8) year old cohort

The 5-year-old cohort was observed 3 years apart and also presented an incidence proportion of 0.8 and an increase in prevalence from 81 to 92% by age 8. This age group differs in that during the period the upper labial sextant would have transitioned from primary incisors to permanent incisors in most children. Nevertheless, an upper labial sextant BEWE score of 3 in this age group still increased from 19 to 29% indicating involvement of the newly erupted permanent incisors or further deterioration of the primary canines. In this study erosive tooth wear was recorded for the whole dentition with no distinction made between primary and permanent teeth. In accordance with the literature that reports a greater progression for erosive lesions in primary teeth [22, 48] this study highlights the difference in rate of wear between the primary and permanent dentition. While 71% of the 3-year-old cohort showed signs or erosive tooth wear on a primary dentition erupted in the mouth for 2 years, permanent molars present in the (5–8)-year-old cohort for 3 years showed little clinical signs of erosive wear.

#### Risk predictive model—age 5

The final risk model for this age cohort included three erosive wear related predictors: baseline BEWE score (p = 0.002), gender (p < 0.001),consumption of iced tea (p = 0.003) and dry mouth (p = 0.029). This is a model with an AUC of 0.9 which classifies it as statistically excellent model having a sensitivity of 95% as defined in previous literature [30]. Interestingly, the risk predictive model for the 5-year-old cohort was driven by a very different set of variables when compared to that of the 3-year-old cohort.

Yet again baseline BEWE cumulative score remained an important predictive factor. The consumption frequency of ice tea, which is classified as a soft drink, was influential enough to render it one of the determinants in the development of the risk predictive model at age five. This is in accordance with previous research which states that the consumption of soft drinks by 5–7 year old children had a significant impact on tooth wear [15] and that a higher frequency of soft drink intake (more than once daily) presented a significantly greater risk of erosive wear in 3–4 year old children [49]. The consumption of ice tea could be a reflection of the family environment and the mother’s educational level, as identified previously that children exposed to a poor diet and unhealthy weight tend to derive from lower socio-economic groups [50].

Another predictive component of this model was gender with males being significantly more affected than females. This is again in accordance with previous literature [48, 51]. Males tend to drink more soft drinks than females and tend to favour stronger flavours and therefore more acidic variants of drinks. The emergence of gender in this 5-year-old age group could therefore be also be a reflection of the increased age of the children and the influence of their personal choices of drinks and foods overriding the preferences of their caregivers. The subjective factor of reporting a dry mouth was also strong enough to enter the predictive model. This factor could be a reflection of the differences in salivary flow between those with and without erosive tooth wear [52].

In this study BMI, a reflection of lifestyle/dietary habits and a risk indicator for chronic diseases, resulted as significantly correlated to change in BEWE scores in the (5–8) year old cohort yet failed to be included in the erosion risk model for preschool aged children. Attempts have been previously made to study the link between BMI and oral health findings and an association between BMI and erosion risk has been reported in older children [53].

Subgroups of a population are at higher risk than others of developing behaviours or conditions. Risk predictive models identify these high risk individuals allowing for early diagnosis and the implementation of risk reduction strategies [27] or intervention. This study has identified those risk factors associated with the probability of a preschool aged child already having or developing erosive tooth wear at a later date. The models created by this study are predictive models and maximise the ability to identify high and low risk individuals—maximising sensitivity and specificity [27]. This was achieved by including all potential factors into the equation—demographic, socioeconomic, behavioural, clinical and biological variables—not just modifiable aetiological factors but also those that are immutable to change. Demographic factors are immutable to change [27] but help to identify target groups. This study has shown that the risk models for the 3 and 5-year-old children, despite being so close in age, are governed by very diverse variables. This leads to the rejection of the Null Hypothesis that stated that there is no difference between risk predication models developed for a 3-year-old and 5-year-old population.

The limitations of this study included participant loss due to non-compliance, absenteeism or lack of parental consent. Additionally, much of the data concerning oral hygiene practices and dietary habits were derived from a questionnaire which may be influenced by bias and the reluctance to disclose full information. The questionnaire could have omitted relevant food items, although the option to include ‘other’ food items was provided. A further factor to include in further research would be the influence of parental and child behavioural factors upon dietary choices and oral hygiene practices. Children with a ‘difficult’ temperament are at a higher risk for dental caries [54]. This is due to parents consenting to habits that do not reflect their own parenting style but that are allowed due to the difficult behaviours of the child. Predictor variables that did not correlate significantly with the outcome variable with univariate analysis were removed from the model. This forward selection method used to generate the risk models does not provide for simultaneous assessment of the effects of all variables and may have weakened the model by allowing important predictors to be eliminated.

## Conclusions

This study established risk predictive models for erosive tooth wear in preschool aged children and has identified the different sociodemographic and behavioural risk factors that influence erosive wear at age three and age five. While risk in the 3-year-old cohort is directed mostly by demographic factors of the caregiver, by age five risk is also influenced by the child’s gender and personal dietary choices. Patient care and educational campaigns aiming to reduce erosive wear prevalence therefore need to be age specific and linguistically and culturally adapted in order to be effective.

## Data Availability

The datasets generated and/or analysed during the current study are not publicly available due to the fact that this study formed part of a larger study and third party permissions would be necessary to release the data, however, data are available from the corresponding author on reasonable request.

## Abbreviations

TSL: Tooth surface loss
COVID-19: Corona Virus Disease 2019
BEWE: Basic Erosive Wear Examination Index
ICDAS: International Caries Detection and Assessment System
CPITN: Community Periodontal Index of Treatment Needs
BASCD: British Association for the Study of Community Dentistry
BMI: Body Mass Index
ROC: Receiver Operating Characteristics
AUC: Area under Curve
